# Prospective memories in the wild: Predicting memory for intentions in natural environments

**DOI:** 10.3758/s13421-022-01379-y

**Published:** 2022-12-20

**Authors:** Jan Rummel, Jean-Paul Snijder, Lia Kvavilashvili

**Affiliations:** 1 Department of Psychology, Heidelberg University, Hauptstrasse 47-51, 69117 Heidelberg, Germany; 2 Division of Psychology, School of Life and Medical Sciences, University of Hertfordshire, Hatfield, UK

**Keywords:** Prospective memory, Everyday intentions, Ecological validity, Individual differences, Ecological momentary assessment (EMA)

## Abstract

Prospective memory, the ability to remember an intention at the appropriate future moment, is often investigated in the laboratory to maximize experimental control. However, demands of laboratory prospective memory tasks only partly map onto everyday demands. Therefore, it is an open question whether factors which predict prospective memory in the laboratory also predict prospective memory in the real world. We combined diary and ecological momentary assessment methods to investigate which factors, that have been repeatedly shown to predict prospective memory performance in laboratory tasks, are related to the fulfillment of everyday intentions. Results showed that substantial portions of variance in real-world prospective memory performance could be explained with the factors found to be significant in laboratory. The most powerful predictors were perceived intention importance, the use of external memory aids, delay interval, and conscientiousness. However, some meaningful laboratory predictors (e.g., working memory) played only a minor role in natural environments and a large portion of the variance in everyday intention fulfillment remained unexplained. The results substantially extend the understanding of conditions and personality variables most conducive to remembering intentions, but they also suggest that additional factors influencing real-world prospective memory remain to be discovered.

Episodic memory is usually thought of as a cognitive system allowing humans to preserve information from the past. It has been argued, however, that this system can also be useful for planning and preparing for the future (Baumeister et al., 2016; Klein, 2013; Tulving, 2005). One important future-oriented memory function is remembering to execute previously formed intentions at the appropriate moment (Schacter et al., 2017; Szpunar et al., 2014). This function has been studied under the term prospective memory (PM) by psychologists for more than 3 decades (see Cohen & Hicks, 2017; Rummel & McDaniel, 2019, for recent overviews). Typical examples of PM tasks may include remembering a dentist’s appointment, a work meeting, or a friend’s birthday party, but also more mundane activities such as remembering to close a window before leaving the house or to take one’s medication with breakfast. From these examples alone, it becomes obvious that PM plays a crucial role in everyday life. Studies using ecological momentary assessment (EMA) of thought contents further suggest that a substantial portion of the thoughts we engage in on a daily basis are future oriented (Smallwood & Schooler, 2015), with a considerable amount of these future thoughts being concerned with planning and rehearsing short-term intentions (Anderson & McDaniel, 2019; Gardner & Ascoli, 2015; Warden et al., 2019).

Given the omnipresence of PM demands in our daily activities and thoughts, it is important to understand when and for whom PM is likely to succeed as well as when and for whom it is likely to fail. Most readers will probably agree that one would expect some intentions to be especially likely to be remembered, such as bringing the ring to the wedding as the best man, because they are particularly important. Furthermore, we certainly all know someone who is particularly unreliable when it comes to fulfilling intentions, such as a relative who always forgets to return phone calls or pass on a message. Thus, intuitively, it seems plausible that characteristics of the intention (e.g., its perceived importance), as well as individual dispositions (e.g., a person’s tendency to be conscientious), influence PM successes and failures. Several studies have been conducted to identify intention characteristics that may predict whether an intention is fulfilled or not (such as the intention’s perceived importance; Kliegel et al., 2004). Some studies have also been conducted to test whether certain cognitive abilities (e.g., individual differences in working memory capacity; Arnold et al., 2015) or personality traits (e.g., conscientiousness; Cuttler & Graf, 2007) are predictive of successful intention completion. However, most of these studies have been conducted in the laboratory, experimentally isolating one of the candidate predictors. In the present research, we aimed to test which of the factors identified in the laboratory as PM relevant play a role for everyday PM performance and to investigate the relative contribution of these predictors. These are important questions because predictors of cognitive performance in the real world might not work in isolation (as in the laboratory) but influence and interact with each other. Furthermore, and real-world tasks might even involve different mechanisms than corresponding laboratory tasks (Shamay-Tsoory & Mendelsohn, 2019). In line with these concerns, we recently argued that the cognitive demands of a typical laboratory PM task do not map onto real-world PM tasks one to one (Kvavilashvili & Rummel, 2020; Rummel & Kvavilashvili, 2022).

A common demand of any PM task inside and outside the laboratory is that people holding an intention have to remember to interrupt their ongoing activities at the appropriate moment by themselves (i.e., without an explicit external prompting) to execute the intention in a timely manner (Ellis, 1996; Kvavilashvili, 1987). More than 20 years ago, McDaniel and Einstein (2000) proposed a theoretical framework that was primarily intended to explain how people manage to switch their attention away from their ongoing activity toward the pending intention when the time has come to fulfill it (McDaniel & Einstein, 2000). This multiprocess theory highlights several characteristics of the retrieval situation that are likely to affect PM remembering, such as, for instance, the difficulty or complexity of the currently ongoing task and particularly how difficult it will be, in the context of this task, to notice the appropriate moment for intention completion. However, the authors of the multiprocess framework also argued that certain intention characteristics—namely, those that are likely to affect the perceived importance of a particular intention—will influence the likelihood with which this particular intention will be executed on time. Furthermore, these authors discussed several person characteristics—namely, individual differences in cognitive abilities, in personality, or in the level of involvement in intention-related planning—as being likely to influence PM performance.

The seminal theoretical framework by McDaniel and Einstein (2000), which has been cited more than 1,350 times in the past 22 years, has inspired much empirical research. Most research has been conducted in the laboratory, using a task setting developed by the same scholars (Einstein & McDaniel, 1990). In this task, which can be considered the standard paradigm for studying PM in the laboratory, participants must remember to execute an intention (usually to press a special key) whenever a target stimulus (e.g., the word *tortoise*) is presented in the context of some ongoing task (e.g., making category judgments). The number of times the intention is executed is usually taken as a measure of PM performance. This task setting is optimally suited to impose manipulations during intention retrieval, such as manipulations of ongoing-task difficulty (Marsh et al., 2002) or target-noticing difficulties (Scullin et al., 2010), and has been extensively used for this purpose. However, it has also been used to investigate how intention and person characteristics affect PM performance.

McDaniel and Einstein (2000) suggest that the perceived importance of and the difficulties associated with remembering a particular intention, are important characteristics which will affect the likelihood of intention fulfillment. In line with these theoretical ideas, it has been repeatedly shown that intentions that were introduced as being important or for which personal importance was increased by associating their fulfillment with a reward are particularly likely to be executed (Cook et al., 2015; Kliegel et al., 2004; Walter & Meier, 2016). Similarly, intentions that were introduced as being of value to others (e.g., to a student experimenter who requires data for their thesis) have been shown to be more likely to be executed than those that are not of social value (Brandimonte et al., 2010; Walter & Meier, 2017). Furthermore, it has been shown that intentions that have been off-loaded from memory (e.g., by using external memory aids) are more likely to be executed than those that must be recalled from memory without a memory aid (Gilbert, 2015; Gilbert et al., 2020), highlighting the role that intention-induced memory load may play for timely execution of intentions (McDaniel & Einstein, 2000). Similarly, intentions that have to be maintained over longer time periods are more likely to be forgotten than those that can be executed after a short time period (Martin et al., 2011; Slusarczyk et al., 2018), although empirical evidence regarding the existence and direction of such intention delay effects are mixed both inside (Hicks et al., 2000) and outside the laboratory (Nigro & Cicogna, 2000).

McDaniel and Einstein (2000) further suggest that certain person characteristics will influence the likelihood of successful PM remembering. In their view, the most promising candidate factors in this regard are individual differences in cognitive capacities, as indexed, for instance, by working-memory capacity or one’s tendencies to mentally revisit pending intentions, as well as some PM-specific personality traits, such as individual differences in conscientiousness. In line with this reasoning, high working-memory capacity as well as high conscientiousness levels have been found to regularly go along with higher intention-execution rates in the laboratory (Arnold et al., 2015; Cuttler & Graf, 2007; Smith et al., 2011; Uttl et al., 2013). In line with the idea that dispositions to think less or more about unfulfilled intentions affects intention fulfillment, diary studies have found positive relationships between the likelihood of engagement in intention-related thoughts and intention fulfillment (Kvavilashvili & Fisher, 2007; Mason & Reinholtz, 2015; Szarras & Niedzwienska, 2011), a finding also observed in some recent laboratory studies (Rummel et al., 2017; Seli, Carriere, et al., 2018a; Seli, Smilek, et al., 2018b). Additionally, the scores achieved in questionnaires developed to assess self-reported everyday PM abilities have sometimes, but not always, been shown to correlate with intention-execution rates in the laboratory (Zimprich et al., 2011; but see Uttl & Kibreab, 2011).

Notably, in the laboratory, PM is investigated under controlled conditions and particularly the use of external memory aids (e.g., calendar use or reminder setting) can be prevented or controlled for. However, some everyday PM demands are not well covered by laboratory tasks, which limits their ecological validity (Kvavilashvili & Ellis, 2004; Rummel & Kvavilashvili, 2019). Obviously, pressing a key in response to a stimulus is less complex than are most everyday intentions. Everyday ongoing activities usually do not require continuous responding like most ongoing laboratory tasks do. The time frame for laboratory intentions is often much shorter than the one for real-life intentions. That is, intentions have to be remembered only for several minutes in the laboratory but for hours, days, or weeks in everyday life. Furthermore, laboratory intentions are almost always assigned to participants by the experimenter. Everyday intentions, however, are often self-assigned—that is, participants plan for themselves what they intend to do and when (for a summary of differences between laboratory an everyday PM tasks see Rummel & Kvavilashvili, 2022, Table 2). Taken together, from a theoretical standpoint, it is not self-evident that predictors of laboratory PM play a pivotal role for PM in real-world settings.

Indeed, Unsworth et al. (2012) found that performance in two laboratory tasks of event-based PM did not correlate with self-reported everyday PM performance recorded by participants in a diary, even when a latent factor was extracted from the laboratory tasks. Interestingly, in the same study sample, performance in laboratory retrospective memory and attention-control tasks *did* correlate with the number of recorded retrospective memory and absent-minded attention failures. The lack of a correlation between PM measures in and outside the laboratory is not surprising because, as discussed above, the laboratory PM tasks have been optimized for studying experimental effects on PM and not for assessing general PM abilities. Furthermore, the laboratory PM tasks’ psychometric properties as an individual difference measure have been known to be not optimal for quite some time (Kelemen et al., 2006).

It is important to note that, although one would not necessarily expect laboratory PM performance to be strongly correlated with intention fulfilment in everyday life, laboratory research on PM is ultimately conducted with the aim of understanding how and when people remember their intentions in the real world. Consequently, the laboratory findings should translate to real-world phenomena, at least to some extent (Rummel & Kvavilashvili, 2019, 2022). Although real-world impact is certainly an important benchmark for any laboratory PM effect, and ideas regarding PM functioning are often inspired by real-world phenomena, very few empirical attempts have been made to reconnect laboratory findings on PM predictors to the real world. The only study that we are aware of is a study by Wójcik et al. (2021). This study focused mainly on the characteristics of the intention retrieval situation in real life, such as whether the intention execution was associated with a certain time point (time-based PM) or a certain event (event-based PM), as well as some affect variables (e.g., stress), and demonstrated that a standard laboratory finding of superior performance in event-based tasks was not found in their sample. In fact, the pattern was reversed, with participants reporting that they completed a higher number of their own real-life time-based than event-based intentions formed at the start of each of the 5 consecutive days of the study.

To further address this gap, we conducted a preregistered field study to test whether a selection of intention and person characteristics that are particularly relevant for successful PM performance, as suggested by multiprocess theory (McDaniel & Einstein, 2000) and demonstrated by previous laboratory studies, play a significant role for intention execution in natural environments. Our participants had to list their intentions for the upcoming 5 days and, after 5 days had passed, they had to indicate which of these intentions they had actually completed. Similar ecological approaches have been developed in the 1990s by PM researchers (Ellis & Nimmo-Smith, 1993; Marsh et al., 1998) but have rarely been used since then, although they have been proven to be well suited to render ecological validity to laboratory results (Schnitzspahn et al., 2016; Wójcik et al., 2021).

As potentially relevant intention characteristics, we assessed perceived intention importance, whether the intention was of social value or not, whether or not external memory aids had been used to remember it, as well as how many days had passed since participants listed the intention for the study. As potentially relevant person characteristics, we assessed Big Five personality factors, as well as individual differences in working-memory capacity, self-rated PM abilities (MPMI-s; Rummel et al., 2019) and social-value orientation (Murphy et al., 2011).

Based on previous findings from laboratory studies of PM, predictions for intention-related variables were that intention importance, off-loading, and its social value would all be positively related to intention execution, while the delay interval from listing intentions to the day of their execution would be negatively related to successful intention completion. Predictions for person-based characteristics conscientiousness and openness, working memory capacity, self-rated PM abilities would all be positively related to successful intention completion. Moreover, at least for intentions of social value, social-value orientation was expected to be positively related with intention fulfillment.

An important and additional aspect of the study design was that during the 5 days of the study, we used EMA to probe participants on their thoughts not only to assess the prospective bias in participants’ thoughts documented in several mind-wandering and PM studies (Anderson & McDaniel, 2019; Gardner & Ascoli, 2015), but also to assess their general tendency to engage in updating/refreshing the representations of upcoming PM tasks and plans. To our knowledge, this is the first study that combines the EMA and the method of recording of participants’ pending intentions and their completion rates. Although we were expecting to replicate the prevalence of future-oriented bias in participants’ thoughts, our primary focus was on assessing the hypothesis that participants who reported higher number of intention-related thoughts in general (some of which could have included also previously listed intentions) may be more likely to also report completing the intentions nominated at the start of the study (Day 0, Fig. 1). This prediction aligns well with the pragmatic dual-process account of everyday prospection (Kvavilashvili & Rummel, 2020), which suggests that the primary purpose of everyday prospection is to engage in thinking about upcoming PM tasks in the immediate future rather than thinking about more hypothetical and wishful scenarios. In line with this assumption, we also examined the time frame of reported future thoughts and expected that the most thoughts about future intentions and plans would have a fairly short time frame for execution (the next 24–48 hours).

**Fig. 1:**
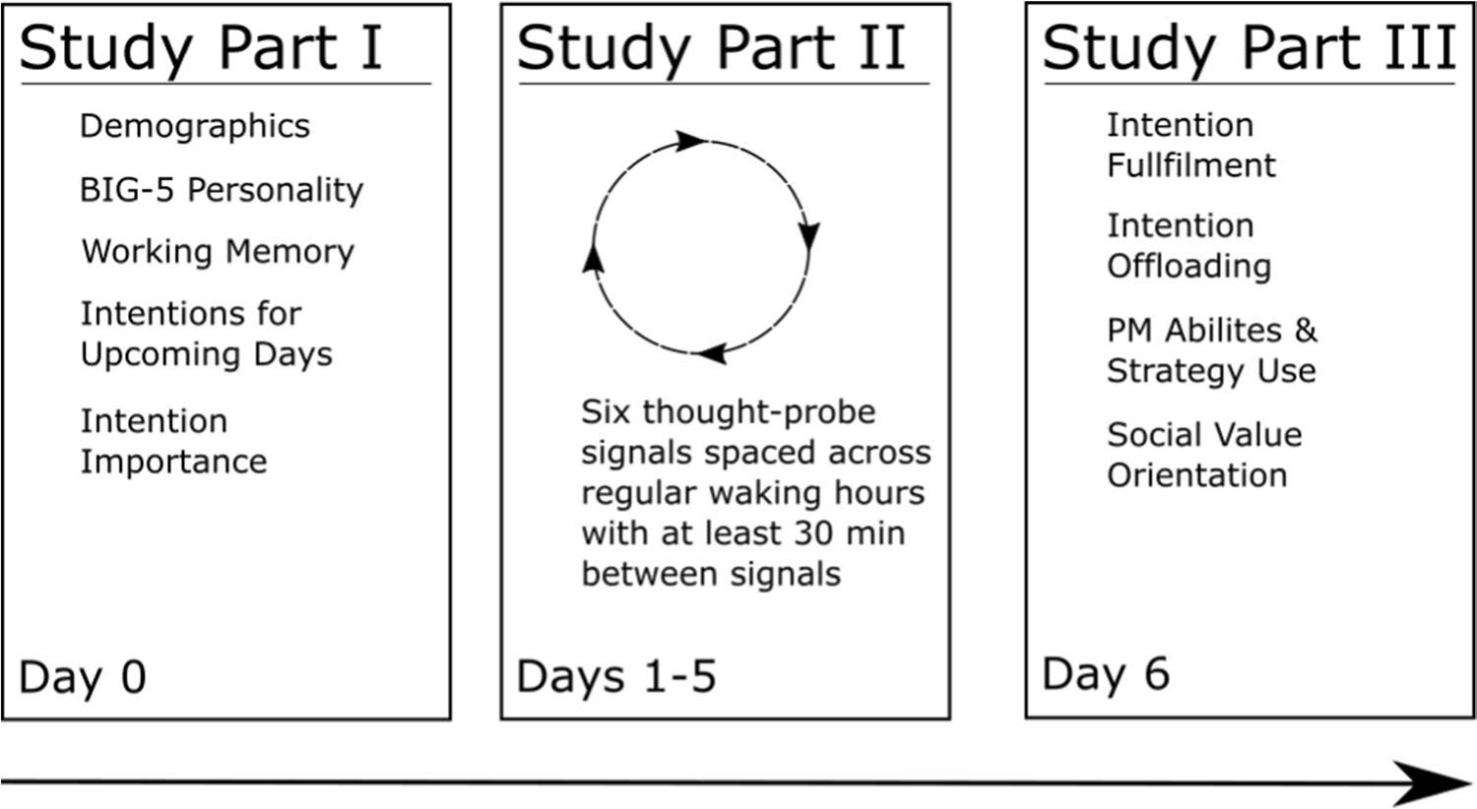
Illustration of three study parts and (online) tasks completed by participants on Day 0, Days 1–5, and Day 6

## Method

Following recommendations by Simmons et al. (2012), across the subsequent Method and Results sections, we report how we determined our sample size and all data exclusions, manipulations, and measures in the study. Due to the COVID 19 pandemic at the time, data for the present study were collected online. This study was preregistered on the Open Science Framework (10.17605/OSF.IO/AUSH2).

### Participants

In line with the preregistration, we aimed to collect data from at least 220 participants, to achieve reliable parameter estimates in our multilevel model analyses (Maas & Hox, 2005). To account for potential exclusions, 259 participants were recruited online from a student participant pool as well as via Facebook groups and mailing lists by a student researcher who used parts of the data for her bachelor’s honor’s thesis.^1^ One participant for whom data recording partly failed and eight participants with less than 10% of correct responses in the working-memory task were excluded from all analyses. Participants of the final sample (*N* = 250) were between 18 and 65 years old, with a median age of *M* = 23 (*SD* = 6.9). Two hundred and one participants (i.e., 76.9%) selected the female gender category, one participant selected the nonbinary category, and the remaining participants selected the male category.

### Prospective memory performance predictors

As intention characteristics, number of days since listing the intention, intention importance, social value, and intention off-loading were considered as potential predictors.

#### Days since listing the intention

On the first day of the study, participants were asked to list their intentions for the next 5 days separately for each day. To indicate how many days had passed between listing an intention and executing it, values from 1 (first day) to 5 (final day) were used. A higher value thus indicates that more days elapsed between intention listing and execution.

#### Intention importance

For each intention participants listed, they were asked to indicate how important they perceived it on a slider scale ranging from 1 (*not at all important*) to 100 (*very important*). Higher values indicate that the intention was perceived as more important.

#### Social value

All intentions were coded by two independent student raters, who were not aware of the study aims, as being either of social value (1) or not (0). For this purpose, raters were instructed to judge each intention description provided by the participants regarding whether its fulfillment or nonfulfillment would have a direct impact on other people or not. For example, “I plan to prepare an assignment for my study group” would qualify as an intention of social value, whereas “I plan to study for my exam” would qualify as an intention not being of social value. Raters were encouraged to make a decision but were allowed to leave out intentions they were indecisive about. With a Cohen’s Kappa of .73, interrater agreement was substantial (Landis & Koch, 1977). For the present analyses, when both raters agreed that an intention was of social value or when one rater thought so and the other one was indecisive, the intention was coded as being of social value. In all other cases, the intention was coded as not being of social value.

#### Intention off-loading

On the last day of the study, participants were presented with all intentions they had listed for completion on the first day of the study. For each intention, participants had to indicate whether they had used an external memory aid (e.g., an automated calendar reminder, a sticky note, or the like) to off-load this particular intention from memory. Off-loaded intentions were coded as 1; not off-loaded intentions were coded as 0.

As person characteristics, Big Five personality factors, working-memory capacity, frequency of intention-related thoughts while the study was ongoing, PM abilities, and, for exploratory purposes, social-value orientation were considered as predictors. We also assessed individual differences in mind wandering behavior with an unpublished questionnaire we recently developed with the primary aim to validate the questionnaire’s structure within the present sample. This questionnaire is designed to measure the frequency of the occurrence of task-unrelated thoughts in everyday life as well as the perceived controllability of these thoughts. As none of the scales of this questionnaire correlated with intention fulfillment rates and as mind wandering is not of primary interest for the present research question, we will not refer to this questionnaire in the Results section.

#### Personality factors

To assess the personality factors (openness, extraversion, agreeableness, conscientiousness, and neuroticism), we used a short form of the Big Five Inventory (BFI-10; Rammstedt et al., 2014). Each factor is assessed with two statements (e.g., *I see myself as someone who does a thorough job*; conscientiousness) which have to be rated on a scale from 1 (*strongly disagree*) to 5 (*strongly agree*). Factor values were calculated by averaging across the responses to statement items after recoding reverse-coded items for each factor. Higher values reflect higher scores on the respective personality factor.

#### Working memory capacity

Working memory was assessed with a variant of the widely used operation-span task (Unsworth et al., 2005). The task was reprogrammed to run on the SoSci survey platform but resembled the task programmed by Unsworth and colleagues. On each trial of this task, participants were presented with a series of letters on the screen one after the other. Between the presentation of two letters, participants decided whether a presented math equation was correct or not (e.g., 5 − 4 = 9). After a set of four to eight letters was presented, participants were asked to recall the letters in the order they were presented. We realized three trials of each set size (4, 5, 6 ,7, 8), for 15 trials in total. The number of letters recalled at the correct positions was used as the working-memory score, following partial-credit unit scoring (Conway et al., 2005). Higher values reflect a higher working-memory capacity.

#### Intention-related thoughts

Individual differences in the occurrence of intention-related thoughts during the intention-fulfillment days were assessed with a thought content survey, similar to the one used by Warden et al. (2019). A signal to fill in the survey was sent out to participants’ smartphones repeatedly—that is, 6 times per intention-fulfillment day (see Procedure section for details). The full survey is provided on the OSF as part of the preregistration (https://doi.com/10.17605/OSF.IO/AUSH2). The survey started with two open-ended questions, first asking participants what they did at the moment they received the survey and second what they were thinking about. The following three questions were multiple-choice questions asking participants about the intentionality of their current thoughts, whether these thoughts were triggered by something in the environment, and about the time perspective (past, present, future, no specific time perspective) of their thoughts. Depending on the answer to the latter question, participants were asked to specify the thought content by choosing from a list of options provided. For example, when participants reported a future-oriented thought, they received the following options to choose from:

- I planned to do something in the future.
- I thought about a future plan I had made earlier.
- I mentally simulated a future event.
- I engaged in fantasies regarding how the future may be.
- I envisioned how I may feel in a future situation.
- I thought about a problem I may face in the future.

A final follow-up question asked participants about the time window of their future- or past-related thoughts—that is, whether they referred to the next/past couple of days, the next/past week, the next/past months, or the next/past years.

The frequency with which each participant selected the options “I planned to do something in the future” and “I thought about a future plan I had made earlier” divided by the total number of survey signals participants actually responded to was used as an indicator for the engagement in intention-related thoughts. Higher scores thus reflect a higher propensity to engage in intention-related thoughts.

#### Prospective memory abilities

PM abilities were assessed with the short version of the Meta-PM Inventory (MPMI-s; Rummel et al., 2019). This questionnaire comprises three scales. The PM ability scale consists of eight items describing typical everyday prospective failures and successes (e.g., *I receive overdue notifications because I forget to pay bills on time.*). The internal and external PM strategy scales consist of seven items each (e.g., *I think of my to-do list while I am busy doing something else, like washing dishes or working out*, or *I keep a calendar with all of my appointments.*). All items are answered on 5-point Likert scales (1 = *rarely*, 2 = *rather rarely*, 3 = *sometimes*, 4 = *rather often*, and 5 = *often*). Mean responses to all items of the respective scale after recoding reverse-coded items were used as indicators for PM abilities and strategy use. Higher scores reflect better PM abilities or a higher propensity to use PM strategies, respectively.

#### Social-value orientation

Individual differences in social-value orientation were measured with the six primary items of the Social Value Orientation Slider Measure (Murphy et al., 2011). For each item, participants needed to make a resource allocation choice from a predefined selection of nine joint payoffs in Euro (e.g., participants had to decide if they wanted to allocate *70 Euros to themselves and 0 Euros to another person*, or *50 Euros to themselves and 40 Euros to another person*, or *30 Euros to themselves and 80 Euros to another person*). Following Murphy et al. (2011), individual social-value-orientation indices were calculated as the inverse tangent of the ratio between the mean allocation to the other person and the mean allocation to oneself (both centered). Higher scores reflect a stronger social-value orientation.

### Procedure

The study was programed in and hosted on the open-source SoSci Survey platform (Leiner, 2021). The study consisted of three parts (see Fig. 1). Participants received a study invitation link and, after clicking on it, were forwarded to the first study part. For this part, participants had to first work on a demographic questionnaire, the Big Five questionnaire, and the operation-span task. Next, participants had to list two to five intentions for each of the upcoming 5 days.^2^ Additionally, they had to rate the importance of each intention. Participants were then asked to provide their email address and mobile number. The latter was used for the second (EMA) part of the study. For the EMA part, participants further indicated when they would usually get up and go to bed (i.e., their regular waking hours). The second study part started the next day. From this day onwards, on each of the 5 days, participants received six thought-probing signals (i.e., text messages with a link to the thought-probe survey) on their mobile phones. To optimally schedule these signals for each participant, individual’s waking hours were divided into six equal time blocks. Within each block, a signal was presented, with the exact time for each signal being randomly selected while ensuring that consecutive signals were spaced at least 30 min apart. Participants could respond to the signals immediately after receiving them (and had been instructed to answer them directly), but survey links remained valid for 30 min. After 5 days, participants received a final signal with another link which remained valid for 24 hours. By clicking on this link, participants started the third study part. In this part, participants were first prompted with their previously listed intentions and had to indicate which of the intentions they had fulfilled and whether they had used an external memory aid to not forget it (e.g., put it in their calendar, set a reminder for it, wrote it on a sticky note). Then, participants filled in the PM, the mind wandering, and the social-value-orientation questionnaires. Participants were finally asked whether they agreed to be contacted by a cultural anthropologist for a follow-up qualitative interview (data not reported). Participants received a debriefing and instructions regarding how to get their participant compensation, on the next day. Participants were compensated with 10 Euros for their participation. Participants who had responded to at least 80% of EMA signals received an additional 20 Euros.

## Results

Data files and analysis codes are provided on the OSF (https://osf.io/aush2/).

### Analysis of thought contents

Thought content analyses were conducted with JASP (Love et al., 2019). On average, participants responded to 23 (*SD* = 4.88) out of the 30 thought probes (range: 9–30). Most reported thoughts, that is 47% (*SD* = 17), were classified as being concerned with the here and now (i.e., present oriented) and 15% (*SD* = 13) of thoughts were classified as having no clear time orientation. The proportion of thoughts classified as being past-oriented was only 6% (*SD* = 7), whereas the proportion classified as being future oriented was 32% (*SD* = 15). Thus, there was a clear prospective bias in terms of more future-oriented than past-oriented thoughts, *t*(250) = 23.26, *p* < .001, *d* = 1.468.

For the present investigation of real-world prospective memories, future-oriented thoughts were of most interest. Only two participants did not report any future-oriented thoughts. For the remaining participants, 47% (*SD* = 28) of their future-oriented thoughts occurred spontaneously, and 48% (*SD* = 29) occurred deliberately, with only 5% (*SD* = 12) of thoughts not being classified as either spontaneous or deliberate. Of the future-oriented thoughts, 48% (*SD* = 27) were classified as being internally triggered, and 35% (*SD* = 24) as externally triggered. For the remaining 18% (*SD* = 20) of thoughts, no clear origin was indicated. In terms of the time window, 59% (*SD* = 27) of the future-oriented thoughts referred to the next 24 hours, 8% (*SD* = 12) to the next 3 days, 10% (*SD* = 16) to the next week, 18% (*SD* = 22) to the next months, and only 5% (*SD* = 9) to the next year and beyond. The patterns of future-oriented thoughts are well in line with previous findings by Warden et al. (2019). Descriptive values for the content classification of future-oriented thoughts are displayed in Table 1.

**Table 1:** Absolute and relative proportions of future-oriented thought contents

	*M*absolute	*SD*	*M*relative	*SD*
I planned to do something in the future.	7	7	25	21
I thought about a future plan I had made earlier.	7	7	20	20
I mentally simulated a future event.	9	8	29	23
I engaged in fantasies regarding how the future may be.	4	5	13	16
I envisioned how I may feel in a future situation.	2	3	5	9
I thought about a problem I may face in the future.	3	4	9	14

All means represent percentage scores. Absolute means indicate proportions of the respective future-oriented thoughts relative to the total amount of thoughts reported; relative means indicate proportions of the respective future-oriented thoughts relative to the amount of future-oriented thoughts reported

When considering each type of future thought listed in Table 1, mental simulation of future events were the most frequently reported (29%). However, as predicted, a large percentage of all future-oriented thoughts (45%) were concerned with either planning or rehearsing intentions, when these two categories were combined (i.e., 14% of all thoughts reported).

### Analysis of predictors of successful intention completion

Analyses were conducted in R (R Core Team, 2021). Using the lme4 package (Bates et al., 2015), we specified generalized linear mixed-effects regression models, using a logit link function, with the binomial intention-fulfillment variable (0 = not fulfilled; 1 = fulfilled) as the outcome variable. In this model, intentions were nested in participants, so that intention characteristics varied on the within-subjects level and person characteristics varied on the between-subjects level. We allowed for a random intercept on the between-subjects level, to account for individual differences in scale use, while all predictor variables were considered as fixed-effect variables. To identify significant predictors, we used the Wald’s chi-squared test and applied a conventional alpha level of .05. Means and standard deviations of all predictors are displayed in Appendix Table 3. Model statistics and Wald test results for all model parameters are displayed in Appendix Table 4.

In line with the preregistration, in a stepwise fashion, we first specified a model that only featured intention characteristics as predictors (i.e., within-subjects-factors-only model). We included intended completion day (1 to 5), intention importance (1 to 100; person-mean centered), social value (0 = not of social value; 1 = of social value), and intention off-loading (0 = not off-loaded, 1 = off-loaded) to predict intention fulfillment likelihood. The within-subjects-factors-only model explained additional variance compared with a null model with a random intercept only, χ^2^(4) = 250.31, *p* < .001. Follow-up Wald tests showed that all considered intention characteristics were significant predictors of successful intention completion.

Next, we additionally considered different sets of person characteristics (i.e., between-subjects factors) to test which individual difference variables would explain variance in intention completion over and above the intention characteristics. First, we specified a model that contained all Big Five personality factors in addition to the within-subjects factors. This model explained more variance than the within-subjects-factors-only baseline model, χ^2^(5) = 31.25, *p* < .001. According to the corresponding Wald tests, only conscientiousness and openness were significant predictors of intention completion. A model with working-memory capacity as a between-subjects factor did not explain more variance than the baseline model, χ^2^(1) < 1, *p* = .564, and the Wald test for working-memory capacity was not significant. A model considering the interaction between intention off-loading and working-memory capacity in addition to working-memory capacity also did not explain more variance than the baseline model, χ^2^(2) = 3.86, *p* = .145. Next, when comparing a model containing the frequency of intention-related future thoughts during the 5 study days as a between-subjects factor to the baseline model, results indicated a significant increase in explained variance, χ^2^(1) = 5.64, *p* = .018. Results of the Wald test for intention-related thoughts confirmed the significance of this predictor. A model with PM ability as well as internal and external strategy use, assessed with the MPMI-s, explained more variance than the baseline model, χ^2^(3) = 17.30, *p* < .001. Follow-up Wald tests identified PM ability as the only significant between-subjects factor in this model. Finally, we specified a model with social value orientation as a between-subjects factor. This model did not explain more variance than the baseline model, χ^2^(1) = 2.23, *p* = .135, and the Wald test for social value orientation was not significant. As one could assume social-value orientation to be a particularly strong predictor for completing social intentions, we also included a cross-level interaction between social value orientation and social value of the intention as an additional factor. This model, however, did not explain more variance than the baseline model with within-subjects factors only, χ^2^(2) = 2.38, *p* = .304.

In the final model, in addition to the within-subjects factors of the baseline model, we included those between-subjects factors, which had turned out to be meaningful predictors in the separate models, to test whether each of them would account for unique variance in intention completion. This final model explained more variance than the within-subjects-factors-only baseline model, χ^2^(4) = 37.89, *p* < .001. As evident from Table 2, all within-subjects as well as the four between-subjects factors (i.e., conscientiousness, openness, frequency of intention-related thoughts, PM ability) included in the final model remained significant when the variance portions explained by the other predictors were controlled for.

**Table 2:** Predictors of successful intention completion included in the final model

	*b*	*SE*	*b/SE*	*dom*	*rank*	χ^2^(1)	*p*
Within-subjects factors							
Days since intention listing	−0.16	0.03	−5.36	.12	3	28.74	<.001
Intention importance	0.02	0.002	10.53	.43	1	110.91	<.001
Social value of intention	0.28	0.12	2.27	.04	5	5.17	.023
Intention off-loading	0.87	0.11	7.60	.27	2	57.87	<.001
Between-subjects factors							
Conscientiousness	0.23	0.08	2.88	.05	4	8.27	.004
Openness	−0.21	0.08	−3.02	.03	7	9.09	.003
Intention-related thoughts	1.29	0.64	2.02	.02	8	4.07	.044
PM abilities	0.27	0.12	2.36	.04	6	5.58	.018

*PM* prospective memory; *dom* standardized dominance values for each predictor reflecting their relative contribution to the change in McFadden’s pseudo *R*^2^ from the null to the final model; *Rank* importance rank order of predictors according to their relative dominance. The final model had 10 parameters, AIC = 3,467, deviance = 3,447

To quantify the relative importance of each predictor in the final model, we also conducted a dominance analysis with the domir package (Luchman, 2022). As a measure for the variance explained by the predictors, we used the change in McFadden’s pseudo *R*^2^ (McFadden, 1973) from the null to the final model to account for the fact that our criterion was binary (for this modification of the original package code, see https://osf.io/aush2/). Standardized dominance values and the rank order of predictors are displayed in Table 2. Dominance values indicate that intention importance and intention off-loading were the predictors that explained most variance in the likelihood of intention completion. The number of days that had passed since the intention was listed was also a relatively important predictor of intention execution in the final model. Social value of the intention, conscientiousness, openness, and PM abilities, as well as the frequency with which participants engaged in intention-related future thoughts during the 5 days of study each contributed less than 10% of the total variance explained by the final model.

**Fig. 2:**
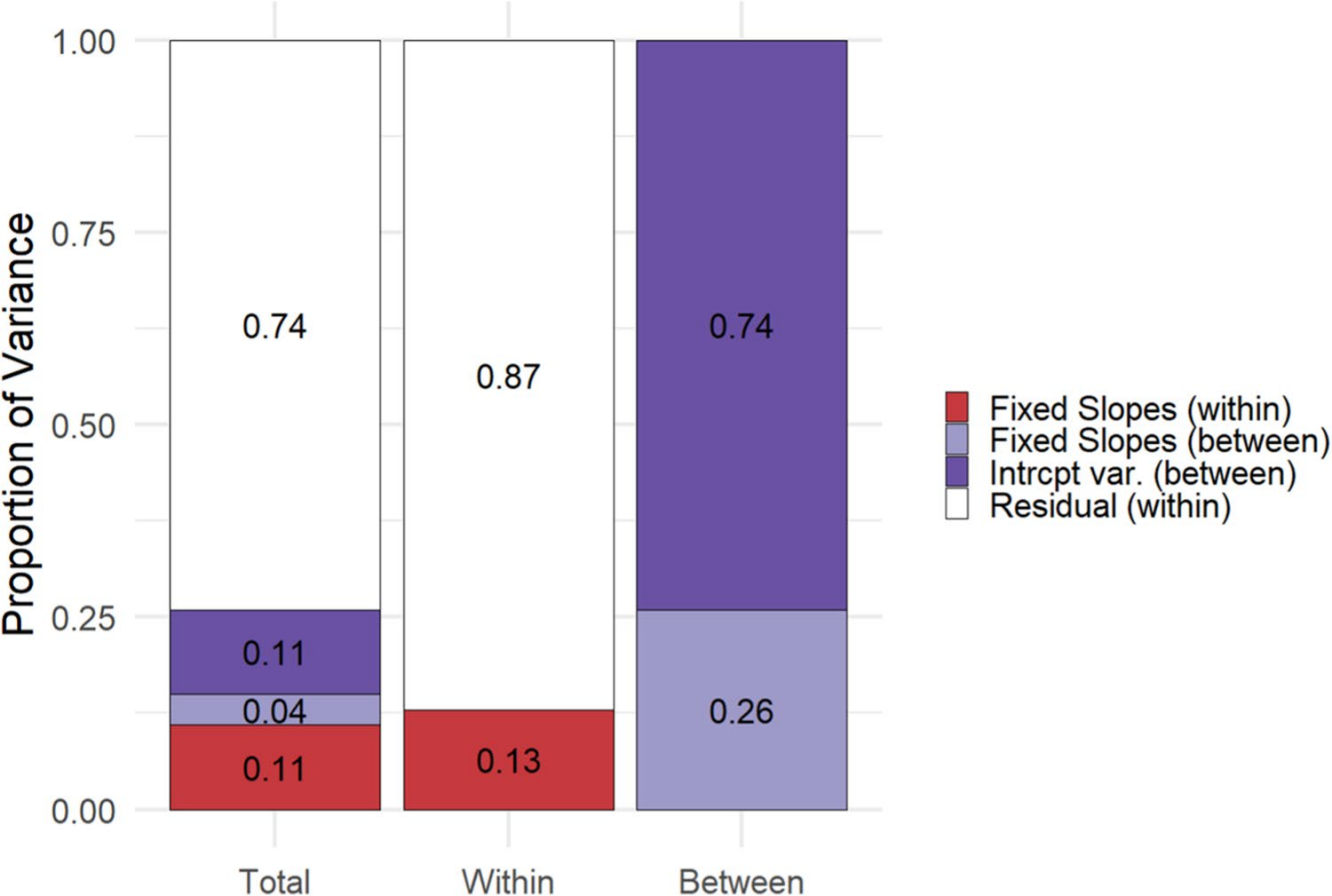
Proportions of explained variance in total, within-subjects, and between-subjects. *Note.* Total = total variance; Within = within-subjects variance; Between = between-subjects variance. The red segments reflect the variance portions explained by the intention characteristics; the lighter purple segments reflect the variance explained by the person characteristics. The darker purple segments reflect the variance attributed to the random intercepts (i.e., between-subjects variance that could not be explained). (Color figure online)

To quantify and illustrate the level-specific and total variance explained by the predictors included in the final model, we used an approach proposed by Rights and Sterba (2020) implemented in the r2mlm package (Shaw et al., 2020). As illustrated in Fig. 2, all predictors together explained about 26% of the total variance in the likelihood of intention completion. The considered intention characteristics explained 13% of the within-subjects variance and the considered person characteristics explained 26% of the between-subjects variance. Thus, although the considered predictors were all significant and explained substantial variance portions, large portions of variance, especially on the individual intention (within-person) level remained unexplained.

## Discussion

Remembering to fulfill intentions at the appropriate future moment is crucial for everyday living. It is thus important to test whether variables that affect PM in the laboratory also impact PM in natural environments. Although insights regarding how the retrieval situation affects real-world intention fulfillment have been gained from using more naturalistic PM tasks in field experiments (e.g., asking participants to make a phone call; Kvavilashvili & Fisher, 2007) and studying participants’ own self-assigned intentions (see Schnitzspahn et al., 2016; Schnitzspahn et al., 2020), the present study is among the first to examine a variety of intention and person characteristics as predictors of participants’ abilities to fulfill their own everyday intentions (see also Wójcik et al., 2021).

Building on previous laboratory findings, our results provide new evidence supporting the significance of most of the candidate intention and person characteristics hypothesized as relevant for successful PM performance within the multiprocess theory by McDaniel and Einstein (2000). Perceived intention importance, the employment of external memory aids, and the intention’s social value were all relevant factors predicting whether everyday intentions had been completed or not. Of those, the first two predictors were also the most important ones overall. Additionally, the number of days between listing the intention for the present study and actually executing them negatively affected intention fulfillment. This may not be surprising as forgetting of an intention (as of any other memory content) can be expected to be a function of the time which has passed since having thought of it for the last time. With the present study design, in which participants had to report their self-assigned intentions rather than getting new intentions assigned by the experimenter, we do not know when participants actually formed their intention. Thus, it may be that some of the intention listed on Day 0 were already pending for quite some time. Nevertheless, our finding nicely parallels with laboratory findings of decreased PM with increased time lags between intention formation and execution (36 versus 6 min; Martin et al., 2011).

Individual differences in conscientiousness and self-reported PM abilities both explained unique variance in intention fulfillment. These results are well in line with the literature on individual differences in PM and demonstrate that the relationships observed under controlled laboratory conditions translate to natural environments (Cuttler & Graf, 2007; Kliegel & Jäger, 2006).

Notably, two findings were not in line with previous research. First, the Big Five factor openness was negatively related to intention fulfillment. As this is at odds with previous laboratory findings and questionnaire studies, which showed no or only small positive relationships between these two variables (see Uttl et al., 2013, for a meta-analytic overview), future research is necessary to clarify whether the present finding replicates in other field settings and within other samples. A second, more thought-provoking finding is the complete absence of a relationship between intention fulfillment and working-memory capacity, even when intention off-loading was controlled for. A positive relationship between these two constructs has been found consistently in laboratory studies (Arnold et al., 2015; Ball et al., 2022; Brewer et al., 2010). So, how can this disparity between laboratory findings and our field study be explained?

Brewer et al. (2010) showed that working-memory capacity was not related to PM performance when the retrieval of intended actions was cued by an easily noticeable event. Despite the suggestions in the literature that everyday PM tasks may be relying predominantly on spontaneous retrieval processes (Kvavilashvili & Fisher, 2007), it seems unlikely, however, that the majority of the self-assigned intentions in the present study were associated with such easily noticeable cues stimulating spontaneous retrieval. One might therefore speculate that the high correlation between working memory and PM observed in the laboratory is partly due to shared method variance, as both constructs are typically assessed with basic cognitive tasks that require shifting between two task goals (i.e., the processing and memory-storing components of a complex span tasks and the ongoing and PM task components, respectively). Besides this methodological issue, however, the regularly shorter time frames in laboratory PM tasks as compared with real-life tasks likely contributed to the inconsistent results. Working memory, as the ability to maintain information active over relatively short periods of time (e.g., Cowan et al., 2005), probably plays a more important role for PM in situations in which intentions need to be maintained for several minutes (as with most laboratory intentions) rather than several days (as with many everyday intentions). Future laboratory studies should thus also realize longer delays between intention formation and execution (e.g., 24 or 48 hours) to evaluate the PM–working-memory relationship (Scullin & McDaniel, 2010).

Another novel and interesting set of findings refers to the nature and frequency of future-oriented thoughts recorded by participants. In addition to finding a prospective bias in recorded thoughts (Stawarczyk et al., 2011; Warden et al., 2019), the results showed that the majority of future-oriented thoughts (59%) referred to future events taking place within the next 24 hours. This focus on the immediate future is perhaps not surprising given that as many as 45% of future thoughts referred to instances when participants were either forming new PM tasks or mentally updating previously formed intentions. These findings are largely comparable with the results of two previous EMA studies, in which participants were *not* asked to list their intentions before thoughts were collected (Anderson & McDaniel, 2019; Warden et al., 2019). Thus, our findings are unlikely to merely be a reactive effect of the intention-listing instructions we used. Importantly, our novel finding is that those participants who engaged more in intention-related thoughts during the study days were found to fulfill more intentions that they nominated at the start of the study, even after controlling for conscientiousness and PM abilities. In the context of other predictors, however, the relative importance of intention-related thoughts as a predictor of successful completion of self-nominated PM tasks was admittedly rather low. However, in our design, we were not able to directly link a recorded future thought to particular intentions nominated at the start of the study (but see Kvavilashvili & Fisher, 2007, for a study in which thoughts were linked to an experimenter-assigned intention). Thus, our study probably underestimated how much intention fulfillment can benefit from a high propensity to think about one’s intentions. Taken together, our findings emphasize the adaptive value of intention-related future thoughts (Klein, 2013; Kvavilashvili & Rummel, 2020) and provide further empirical support for the pragmatic dual-process theory of everyday prospection (Baumeister et al., 2016; Kvavilashvili & Rummel, 2020). Clearly, future studies need to replicate and extend the current findings by examining not only a positive relationship between the propensity of intention-related future thinking and successful PM remembering, but also establish whether such general propensity is a stable individual difference characteristic.

In our analyses, we followed a preregistered analysis protocol and thus, with very few exceptions, specified models testing only for main effects of the selected predictors on intention fulfillment. Nevertheless, we believe that the present data set, which we share on the OSF, allows for many interesting additional exploratory tests of higher-order interaction effects between the factors we assessed and we encourage our readers to use the data for this purpose.

It is important to note that we were able to explain roughly 26% of the total variance in intention fulfilment rates with the factors we considered. Although the contribution of many of these factors was rather small, these results should be still considered substantial (Funder & Ozer, 2019), particularly for a field study in which many environmental (nuisance) factors have to remain uncontrolled. Nevertheless, the present results also suggest that additional factors are most certainly relevant for remembering PM tasks in natural environments. One set of factors highlighted with the multiprocess framework as particularly relevant was not covered by the present study design. That is, the cognitive demands imposed by the prospective memory retrieval situation. As these factors likely explain additional variance in intention fulfillment, an important endeavor for future research will be to come up with clever field-study designs that allow to additionally assess the cognitive demands during intention retrieval. Another important set of factors to be considered may be personal affective states. For example, state fluctuations in individual stress levels have been shown to negatively relate to intention fulfillment in previous ecological momentary assessment and diary studies of PM (Ihle et al., 2012; Wójcik et al., 2021). Interestingly, stress manipulations do not seem to affect PM performance in the laboratory (Möschl et al., 2017)—another instance where divergent PM results have been obtained inside and outside the laboratory. All in all, although the present results identified a set of intention and personality characteristics that are important predictors of everyday prospective memory, characteristics of the retrieval situation should be certainly considered as additional predictors in future studies for a complete picture.

## Conclusion

In line with the theoretical considerations made by McDaniel and Einstein (2000), results of the present study demonstrate that intention characteristics, such as perceived importance of intentions as well as individual differences in person characteristics, such as conscientiousness, prospective memory abilities, and the level of engagement in intention-related future thinking, explain variance in natural intention fulfillment. These factors—which have mostly been studied in isolation and within the laboratory so far—explain significant portions of both the within- and between-subjects variance in the likelihood of successful intention completion. However, substantial portions of variance remained unexplained in the present study, which may not be surprising for multidetermined behavior, such as intention execution in real-world settings. Although future research is necessary, the present results are an important step towards a better understanding of when, under which circumstances, and by whom intentions will be remembered. Additionally, we hope the present study demonstrates how laboratory findings can be reconnected to real-world phenomena by combining diary and ecological-momentary-assessment methods (Rummel & Kvavilashvili, 2019). In doing so, we hope to gain the best from two worlds—namely, strict experimental control within the laboratory and high ecological validity in the natural environment—an approach that might also be of interest for other areas of cognitive psychology.

## Appendix

Appendix Tables 3 and 4

**Table 3:** Descriptive statistics all within- and between-subjects predictors

	*M*	*SD*	Range	Description
Within-subjects predictors				
Intention importance	74.71	24.62	1–100	Assessed on a Likert scale ranging from 0 to 100
Social value of intention	.19	.39	–	Coded as 1 = of social importance; 0 = not of social importance
Intention off-loading	.32	.47	–	Coded as 1 = off-loaded; 0 = not off-loaded
Between-subjects predictors				
Conscientiousness	3.50	.89	1.5–5.0	Assessed on a Likert scale ranging from 1 to 5
Openness	3.82	.95	1.0–5.0	Assessed on a Likert scale ranging from 1 to 5
Agreeableness	3.29	.82	1.5–5.0	Assessed on a Likert scale ranging from 1 to 5
Extraversion	3.28	1.01	1.0–5.0	Assessed on a Likert scale ranging from 1 to 5
Neuroticism	3.30	.99	1.0–5.0	Assessed on a Likert scale ranging from 1 to 5
WMC Accuracy	.80	.14	.12–.99	Assessed as proportion correct
Intention-related thoughts	.14	.10	0–.56	Assessed as proportion scores
PM abilities	3.86	.61	1.5–5.0	Assessed on a Likert scale ranging from 1 to 5
PM strategies internal	3.42	.68	1.7–5.0	Assessed on a Likert scale ranging from 1 to 5
PM abilities external	3.68	.74	1.7–5.0	Assessed on a Likert scale ranging from 1 to 5
Social value orientation	.65	.16	.20–1.15	Assessed as outcome allocation to others/ allocation to oneself ratio score

Within-subjects predictor means were calculated across all within-subjects observations

**Table 4:** Model statistics and Wald test results for all model parameters separate for all models

	χ^2^(1)	*p*	AIC	*n*-par	dev
Null model			3,739	2	3,735
Baseline model			3,497	6	3,485
Within-subjects factors					
Days since intention listing	28.76	<.001			
Intention importance	110.76	<.001			
Social value of intention	5.78	.016			
Intention off-loading	59.49	<.001			
Big-5-only model			3,476	11	3,454
Within-subjects factors					
Days since intention listing	29.08	<.001			
Intention importance	111.47	<.001			
Social value of intention	5.26	.022			
Intention off-loading	57.44	<.001			
Between-subjects factors					
Conscientiousness	18.17	<.001			
Neuroticism	<1	.473			
Extraversion	<1	.478			
Agreeableness	2.91	.088			
Openness	8.41	.004			
Working-memory model			3,498	7	3,484
Within-subjects factors					
Days since intention listing	28.81	<.001			
Intention importance	110.79	<.001			
Social value of intention	5.79	.016			
Intention off-loading	59.13	<.001			
Between-subjects factors					
Working memory	< 1	.563			
Intention-related-thoughts model			3,493	7	3,479
Within-subjects factors					
Intention completion day	28.65	<.001			
Intention importance	110.75	<.001			
Social value of intention	5.68	.017			
Intention off-loading	58.65	<.001			
Between-subjects factors					
Intention-related thoughts	5.75	.017			
PM-abilities-and-strategy-use model			3,486	9	3,467
Within-subjects factors					
Days since intention listing	28.64	<.001			
Intention importance	109.80	<.001			
Social value of intention	5.54	.019			
Intention off-loading	60.76	<.001			
Between-subjects factors					
PM abilities	17.44	<.001			
Internal strategy use	<1	.926			
External strategy use	<1	.859			
Social-value-orientation model			3,497	7	3,483
Within-subjects factors					
Days since intention listing	28.63	<.001			
Intention importance	110.62	<.001			
Social value of intention	5.87	.015			
Intention off-loading	60.36	<.001			
Between-subjects factors					
Social value orientation	2.28	.131			

*n*-*par* number of parameters; *dev* deviance value

## Footnotes

1 We thank Inga von Kortzfleisch for her help with the data collection. Following university regulations, Inga analyzed her data on her own and wrote her unpublished thesis in German independently of the present manuscript.

2 We restricted the number of intentions to five to obtain comparable numbers of observations per participants and to ensure that the pattern of results was not influenced disproportionally by few participants listing a very large number of intentions.
